# Fallacies and Facts

**Published:** 1915-09

**Authors:** H. E. Bliler

**Affiliations:** Chicago, Ill.


### FALLACIES AND FACTS.

BY H. E. BLILER, D.D.S., CHICAGO, ILL.

Reverence for antiquity is responsible for the fact that many greatly overestimate the enlightenment of the savants of antiquity. The most renowned scholars of ancient times were credulous and superstitious to an appalling degree, having implicit confidence in various forms of magic.

Kircher, nearly two and a half centuries ago, expressed the opinion that there were definite micro-organisms to which disease was attributable. The microscope has revealed that all decomposing substances swarm with countless millions of bacteria.

Underlying every science will be found the record of a great amount of observation, verified by clinical activities, from which men have drawn conclusions.

There is no material difference as to the bacteria that cause pyorrhea, carbuncle, appendicitis, headache, swollen glands, faces, etc.,—and most all ailments—for by using thorough and constant eliminative treatment with diet prevention they all disappear, and healthy conditions are obtained. I have personally verified this from long service in clinical activities. Why we have different ailments and local morbid manifestations as above enumerated, all from systemic origin, is because nature is trying to throw off the poisons at the point of least resistance. These poisons are taken up by the blood stream from an unclean alimentary tract, and they consist of putrefactive changes of different kinds of waste products, that are not eliminated through the proper channels. The verdict of science seems to be exactly opposite to popular opinion, and clinical observations frequently controvert laboratory precepts.

Our present knowledge of intestinal putrefactive processes and their related metabolic perversions, which are commonly manifested by toxemias of various degrees of intensity, is such that we no longer regard constipation as other than a condition worthy of the most skillful and painstaking treatment. We know that a stagnated colon bears a causal relation to a great variety of anomalies of metabolism, and that it is a prolific source of functional disturbances.

Billroth found bacteria in small numbers in the upper bowel segments, increasing in the intestines, and the largest number in the fecal repository, or colon. The number found in the whole alimentary tract is almost beyond computation in normal as well as abnormal conditions.

As to the present notion that pyorrhea can be cured by local and internal medication with the new specific emetin, I would cite two opinions that may be of value in considering this subject.

Dr. Valadier, *Dental Review*, January, 1915, says that several weeks ago he had received a paper from Philadelphia giving him the results of Dr. Barrett's work on emetin. He immediately called up his collaborators at the Institute and started to make investigations on the same lines. They immediately studied emetin to its full extent and found it was rather a dangerous drug. No instruction being given as to how it was to be used, it was tried on a monkey in which pyorrhea had developed. The emetin was applied to the gums at 4 o'clock in the evening—and the monkey was dead at 9 o'clock next morning.

Dr. Kirk does not regard it proven that emetin is a specific for pyorrhea alveolaris, or even a specific for the destruction of the entamoeba buccalis. It has been shown very definitely by experiments upon dysentery that emetin is not a specific, but that it is also destructive of bacterial forms as well. He is not sure that pyorrhea is due to a specific infection.

I recently received a letter from a pyorrhea specialist in a western city, in which he makes this statement: "I have just read your paper on basic cause and cure of pyorrhea in *Oral Hygiene*, and am greatly interested in it. I was wondering if you would give me further advice as to your treatment. I am getting some fine results with my local treatment alone, using the Carr instruments and methods, but I find I can not stop the flow of pus." This is a sample of many comments I have received and it indicates to me very clearly that pyorrhea in most cases is of systemic origin, for I have controlled the flow of pus in every case, and have not even cleaned the tartar off in many cases. Much damage may be done, and unnecessary inflammation created, by too much instrumentation.

*What we most need is immunity from erroneous impressions and predatory commercialism.*

My treatment, which I have already published several times, is so simple that its efficiency is discredited by those who are unwilling to put it into practice. It consists in a persistent and discriminating use of the old and well known remedy, oleum ricini, made so palatable that any one can take it without nausea or bad effects. The prescription which I recommend is as follows: Oleum ricini, one ounce, taken in eight ounces of foamed root beer, which can be had at any drug store soda fountain. It should be taken as indicated, once or twice a week, to establish good bowel elimination.

